# Supplementary material for: The role of integration host factor in biofilm and virulence of high-alcohol-producing Klebsiella pneumoniae
Source: Microbiol Spectr. 2023 Sep 21;11(5):e01170-23. doi: 10.1128/spectrum.01170-23 (PMC10581059; doi:10.1128/spectrum.01170-23)
Supplement: Table S1 — Bacterial strains, plasmids, and primers used in this study. [file spectrum.01170-23-s0001.pdf]

Supplementary Table S1 Bacterial strains, plasmids and primers used in this study

Strain/ Plasmid	Description	Source/Purpose
K. pneumoniae		
W14	Wild type strain of High-alcohol-producing *K. pneumoniae* isolated from NAFLD&ABS patient	(1)
Δ*ihfA*	W14 deleted of *ihfA*	This study
Δ*ihfB*	W14 deleted of *ihfB*	This study
Δ*ihfA/ihfA*	Complemented *ihfA* mutant with a pGEM-T Easy plasmid	This study
Δ*ihfB/ihfB*	Complemented *ihfB* mutant with a pGEM-T Easy plasmid	This study
**Plasmid**		
pKO3-Km	Gene replacement plasmid derived from pKO3 with an insertion of Km resistance cassette into *AccI* site	(2)
pKO3-*ihfA*	pKO3 derivative, for *ihfA* deletion	This study
pKO3-*ihfB*	pKO3 derivative, for *ihfB* deletion	This study
pGEM-T-easy	Expression vector with an insertion of Km cassette	(Promega, Madison, WI, USA)
**Primer**	**Sequence (5′→3′)**	**Function**
Ko-i*hfA*-Up-F	TCGGTACCCGGGGATCGCGCCTGACCCTGCCCTATC	Construction of *ihfA* deletion
Ko-i*hfA*-Up-R	TCTGTTTGATAAGCTTGGGCTTAAAAGCCGTGTCGAAAACG	
Ko-*ihfA*-Dn-F	CGTTTTCGACACGGCTTTTAAGCCCAAGCTTATCAAACAGA	
Ko-*ihfA*-Dn-R	GGTCGACTCTAGAGGATCGCAACGACAAAGGCTATCAGGAAG	
Ko-*ihfB*-Up-F	TCGGTACCCGGGGATCGCCAACAAAAACATCCACCCGT	Construction of *ihfB* deletion
Ko-*ihfB*-Up-R	CGGGCTTAAAGTGCGGAACTGGCAAGTCTTTCAATCAATTC	
Ko-*ihfB* -Dn-F	GAATTGATTGAAAGACTTGCCAGTTCCGCACTTTAAGCCCG	
Ko-*ihfB* -Dn-R	GGTCGACTCTAGAGGATCGCCCTAATCCCAACATCACCGC	
C-*ihfA*-TeasyF	AATTGGGCCCGACGTCGCATGCTACCGTTGCCAGATGTGTAGAGG	Expression of *ihfA* in HiAlc *Kpn*
C-*ihfA*-TeasyR	TTGGGAGCTCTCCCATATGGTCGACTCAAAGCGTAAAGATTGACAG	
C-*ihfB*-TeasyF	AATTGGGCCCGACGTCGCATGCACTTGACAGATTGCAGGATTCG	Expression of *ihfB* in HiAlc *Kpn*
C-*ihfB*-TeasyR	TTGGGAGCTCTCCCATATGGTCGACAAGGTGCTTTCTTTATCGTCAG	
tdcA-RT-F	AGAAAATCGGCATTGAGCAC	qRT-PCR
tdcA-RT-R	GATCGGCAACGGCACC	

tdcB-RT-F	CGCAACCATCGGCTAAGG	qRT-PCR
tdcB-RT-R	GTCGGAGAATGTTCACGGC	
tdcC-RT-F	TATTGGGGTTGGCATACGC	qRT-PCR
tdcC-RT-R	CGAGGGGCTGAACGGTC	
tdcD-RT-F	ATTGGGCTGCTGATTGCG	qRT-PCR
tdcD-RT-R	GGTGGGATTGGCGAGAAC	
feoA-RT-F	TTAGCCCAGCATACCGTCA	qRT-PCR
feoA-RT-R	TGCAACCGCTTCCAGTTC	
feoB-RT-F	CGTTGAGCGTAAAGAGGGG	qRT-PCR
feoB-RT-R	GTCTGCGAGGAGATGGTGG	
feoC-RT-F	AAACAGCTCAGTGCTCGCC	qRT-PCR
feoC-RT-R	CGCCGCTTTCCCTTCAG	
fhuA-RT-F	CAAAAACTGCTCAGCCAAATC	qRT-PCR
fhuA-RT-R	CTGCTGCCTGTGCGTAGAC	
tssG-RT-F	ATCTTCATCTCTCTCCCTGCG	qRT-PCR
tssG-RT-R	CTGTGCTGCGTCCTCTGG	
hcp-RT-F	GAAAGACGACGGCGGC	qRT-PCR
hcp-RT-R	ACAGGAACGGCGTATGAATAC	
gspE-RT-F	CGGATTTCGCCCACCA	qRT-PCR
gspE-RT-R	GGCATAGGCCAGACCCAG	
tonB-RT-F	TGGAGCAGTAACGGTGGG	qRT-PCR
tonB-RT-R	CCGAAGCGGGAAGTGAAG	
exbD-RT-F	CTTCGCCCACCAGCCC	qRT-PCR
exbD-RT-R	ACCACCGTCTTCTTCCGC	
exbB-RT-F	ACCAACCGCCGCAACG	qRT-PCR
exbB-RT-R	GGAAGCCCGCTCTCTCG	
wzi-RT-F	CAGTTTACCGCATCCTTACCC	qRT-PCR
wzi-RT-R	TGGAACCGACCCTTGGC	
iscR-RT-F	GCGTGAGTCAGGCATTTATCG	qRT-PCR
iscR-RT-R	CCAGGTGGTGGTTATCTGTTAGG	
rcsA-RT-F	TGTTTGTTATCTTTATGTCGCTGG	qRT-PCR
rcsA-RT-R	CATCAAGGTCTTTTGGGGTTA	
galF-RT-F	GCCCATTTTCTTCCCGC	qRT-PCR
galF-RT-R	CTGACCGACGCCATTGC	
mrkA-RT-F	TTCGCTTTCGGCTGAGTG	qRT-PCR
mrkA-RT-R	AACTGGGCGTGAACTGGAC	
mrkB-RT-F	GGTTTTACTGTTCAGGGCTTTAC	qRT-PCR
mrkB-RT-R	CCAGCGGCAGTAGTCTCG	
mrkJ-RT-F	TGATAATCTTTCTATTTCCCCCG	qRT-PCR
mrkJ-RT-R	CCAGGCTTTATGCTTTTCAATC	
mrkH-RT-F	ACAACACCAGTATCATAACCCAAC	qRT-PCR
mrkH-RT-R	AGGCAATGAAAAAGCGTAGC	
rfbA-RT-F	ACCCCAAACCCAGGACAAG	qRT-PCR

rfbA-RT-R	TTTCATTCATCGCTAACGCTC	
rfbB-RT-F	GGTCTGAATACATTTTGCCTGA	qRT-PCR
rfbB-RT-R	TGAATTTTCGGAACTGGGAG	
wbbM-RT-F	CTTGGCGTGCCTATTGGTG	qRT-PCR
wbbM-RT-R	GATGTCGTTAGCGGCTTCG	
rfbD-RT-F	ACACAGGCATTGGCTGATTT	qRT-PCR
rfbD-RT-R	ACTTTTGTCGGTCGTCTTGG	
A7321_RS12650-RT-F	TGACAAACCGCTTACCGCC	qRT-PCR
A7321_RS12650-RT-R	TACATTTCCCGCAATCCCC	
A7321_RS12655-RT-F	CCTTACGCTACTGGGGTGG	qRT-PCR
A7321_RS12655-RT-R	AATGAGCAATACCTTACCTGTTTC	
sdiA-RT-F	GCTTAGTTCAGTCAGCGTTCG	qRT-PCR
sdiA-RT-R	TCAGGGATTTCTCTCTGTCTCC	
lsrA-RT-F	CGGATTTCGGGCAGTTTGT	qRT-PCR
lsrA-RT-R	GCCAGCACGCTCGAGGTAG	
lsrB-RT-F	GCCCTTCGCCTCATACTGG	qRT-PCR
lsrB-RT-R	CAAAATTGCGGTTAACGTCG	
lsrC-RT-F	GCCACCTCTTTGCTTTTGC	qRT-PCR
lsrC-RT-R	TGGTACTGTTCCGCCTTCC	
lsrG-RT-F	CTGCGACATCATCTCCTCCA	qRT-PCR
lsrG-RT-R	AAAACCCGCTTTTTATCTACG	
lsrF-RT-F	CTCAACGAAATAGGTCTTCACAAA	qRT-PCR
lsrF-RT-R	GCAGCGAATACGAACATCAGTC	
lsrR-RT-F	GAACTGCTCGGCGTAACCC	qRT-PCR
lsrR-RT-R	TGCGCTTTTTCTTCTCCCC	
lsrK-RT-F	ATGATGAGCAAATGGCAGACG	qRT-PCR
lsrK-RT-R	TAGGGTGAGGGGGAAGACG	
bcsA-RT-F	AAGGTGGCGTTCCAGTTGT	qRT-PCR
bcsA-RT-R	CCTGAAAGACCCGATGCTG	
bcsB-RT-F	GCAGGTTGTTGAGGAAGGTG	qRT-PCR
bcsB-RT-R	CTCTATCTGTGGGATGGAGAGAC	
bcsC-RT-F	CGACAACATTGAAGCCCATC	qRT-PCR
bcsC-RT-R	GCGGCAGTAAAAATCAGACC	
bcsE-RT-F	TGTTTGACACCAACGACGAG	qRT-PCR
bcsE-RT-R	TGTTCACGGACGATGATTTTC	
bcsF-RT-F	CAACTGGTGGTGCTCTGCG	qRT-PCR
bcsF-RT-R	CATTTGTTCGCTTTGCCTTG	
bcsG-RT-F	TTAGCCAGGGGTCGCAAAT	qRT-PCR
bcsG-RT-R	CCATAGCACCAGTAAAACAAAGAAG	
bcsO-RT-F	CCGAAGCGGCAGACACAAC	qRT-PCR
bcsO-RT-R	GATCCTCAATCAGCTAAATCCC	
bcsZ-RT-F	GTCTCTTCAGCGACAATCCG	qRT-PCR
bcsZ-RT-R	GGACGATGAGCAGTGGACC	

fimA-RT-F	GGATGATTGCGACACTACGG	qRT-PCR
fimA-RT-R	GTTTTGCAGGGCCAGAACG	
fimB-RT-F	GACAGTAACCCAATCCCTTTCG	qRT-PCR
fimB-RT-R	TTTTTCCATCAGCCACGCC	
fimC-RT-F	AGGATGTGTGCTTTTCGCC	qRT-PCR
fimC-RT-R	GCATTTTCCACCCATGACTG	
fimD-RT-F	GCGAACATCAGGGGCAGTC	qRT-PCR
fimD-RT-R	CGGTAAGTGGTATCGGCAAAG	
fimE-RT-F	GCAGTTTTATGCCGTTGGG	qRT-PCR
fimE-RT-R	GGCTGGTGTTGATGCTGTCC	
fimF-RT-F	TTTAGCCTGATCGGTGCGG	qRT-PCR
fimF-RT-R	CGTTCCCTGGTTTTTGTAATAGC	
fimG-RT-F	CTATGGCGGTGTGCTGTCG	qRT-PCR
fimG-RT-R	GGGTTTATCGGTCCGTGAATC	
fimH-RT-F	TGACGGCGTTTGAACAGGA	qRT-PCR
fimH-RT-R	GTGCGGCAGAAAGGTCTGG	

REFERENCES

(1) Yuan J, Chen C, Cui J, Lu J, Yan C, Wei X, Zhao X, Li N, Li S, Xue G, Cheng W, Li B, Li H, Lin W, Tian C, Zhao J, Han J, An D, Zhang Q, Wei H, Zheng M, Ma X, Li W, Chen X, Zhang Z, Zeng H, Ying S, Wu J, Yang R, Liu D. 2019. Fatty Liver Disease Caused by High-Alcohol-Producing *Klebsiella pneumoniae*. Cell Metab 30:675-688. 10.1016/j.cmet.2019.08.018.

(2) Link AJ, Phillips D, Church GM. 1997. Methods for generating precise deletions and insertions in the genome of wild-type *Escherichia coli*: application to open reading frame characterization. J Bacteriol 179:6228-6237. 10.1128/jb.179.20.6228-6237.1997.
